# Chloride content in goat milk as a diagnostic marker for subclinical mastitis

**DOI:** 10.5455/javar.2025.l922

**Published:** 2025-06-02

**Authors:** Nadiia Zazharska

**Affiliations:** 1 Faculty of Veterinary Medicine, Dnipro State Agrarian and Economic University, Dnipro, Ukraine

**Keywords:** Chlorosugar number, electrical conductivity, goat milk, settling test, somatic cell count, total plate count

## Abstract

**Objective:**

The purpose of the research was to assess possible diagnostic parameters related to subclinical mastitis in goats.

**Materials and Methods:**

Individual samples of milk from goats were separated into three groups based on the chloride concentration: group I < 70 mmol/l; II group 70–85 mmol/l; III group > 85 mmol/l. The composition of milk was studied, and smears were also made.

**Results:**

When the chloride content in goat milk exceeds 85 mmol/l, the somatic cell count increases by 3.2–5.7 times in relation to milk with a chloride content below 70 mmol/l, depending on the research method used. In the second group, the indicators exceed those of the first group by 2.1–3.8 times. Compared to the second and third groups, the indicator of electrical conductivity in goats of the first group was lower by 13.1% and 31.3%, respectively. Milk from healthy goats, characterized by a chloride content of less than 70 mmol/l, shows a chlorosugar number averaging 5 (ranging from 4.1 to 5.9). In cases where chloride levels in milk exceed 85 mmol/l, the average indicator of the chlorosugar number is 7.2 (from 6.5 to 7.9). If the content of chlorides is > 85 mmol/l, a positive result of the settling test with goat’s milk is noted.

**Conclusion:**

A set of indicators such as chloride content > 85 mmol/l, the number of somatic cells >2 million/ml, chlorosugar number ≥ 7, along with confirmed results in the settling and mastidine tests—can be used as a diagnostic criterion for subclinical mastitis in goats.

## Introduction

For the rural population of some countries, goats are a key source of additional income and an important factor in economic development [1–3]. The popularity of a healthy lifestyle is contributing to increased interest in goat milk, which is characterized by lower allergenicity and significant nutritional value compared to cow’s milk. In dairy goats, subclinical mastitis occurs frequently and leads to a decline in milk quality and yield, causing considerable economic damage [4,5]. According to French scientists, subclinical mastitis affected 20%–50% of goats, based on bulk milk somatic cell averages, as reported in the literature up to 2002 [1]. In Romania, the prevalence of subclinical mastitis was diagnosed as 70.21% in goats from herds with somatic cell averages > 1 million/ml [6]. In western Algeria, subclinical mastitis affected 20.7% of goats based on California mastitis test (CMT) results.

The risk was significantly elevated in goats with poor udder hygiene, housed on unclean farms, with multiple pregnancies, or in late lactation [7]. The incidence rate of subclinical mastitis among goats in China was 45.82% (according to the results of CMT). The causative agents using multiplex PCR analysis were: *Staphylococcus* spp*., Escherichia coli,* and *Streptococcus* spp. [8]. The overall prevalence of subclinical mastitis in goats according to the CMT results in India was 23.04%. The age of the animals, the number of lactations, the period of late lactation, and the season of the year (winter) were classified as risk factors [9]. In Nyeri County, Kenya, clinical mastitis in goats was observed at a rate of 1.1%, compared to 84.7% for subclinical mastitis. The reason for such results was low sanitary conditions for keeping and milking animals. Thirteen causative agents of mastitis were detected from milk samples; the majority belonged to the genera *Staphylococcus, Escherichia*, *Pseudomonas*, and *Enterobacter* [10]. A study in Bangladesh examined the incidence rate of subclinical mastitis in goats and its risk factors. The causative agent of mastitis, *Staphylococcus aureus*, was found in 13% of cases. The shape of the end of the teat and the age of the goats were classified as risk factors for subclinical mastitis [11]. In Siliragung Subdistrict, Banyuwangi District, Indonesia, *S. aureus* was isolated from 25.58% of samples of raw goat milk with a positive CMT result. Of these isolates, 36.36% were resistant to antibiotics [12].

Intramammary infections result in changes to the milk composition, reduce hygienic indicators, and worsen the quality of dairy products made from it. It is obvious that subclinical mastitis is the most common infectious disease in small ruminants. In cows, key parameters for diagnosing mastitis include electrical conductivity and somatic cell count (SCC). Evaluating udder health in goats is more difficult [13].

Swedish researchers assessed SCC both indirectly using the CMT and directly using a portable DeLaval cell counter. 39 (18%) goat milk samples tested positive for mastitis pathogens. The most common pathogen was coagulase-negative staphylococci (72%), followed by *S. aureus* (23%). The SCC, determined using a portable DeLaval cell counter, was significantly associated with increased bacterial contamination (*p* = 0.000). The CMT was associated with the detection of pathogens. SCC results from the CMT correlated well with those measured directly by the DeLaval portable cell counter [14]. Scientists study the correlation between the SCC in milk, bacterial contamination, and the composition of milk [15–17]. Experimental infection of the Zaanen goat’s udder was conducted to assess changes in the milk composition. One group was treated with antibiotics, and the other with an ointment based on natural plant extracts. Other groups were created as controls. Increases in fat, protein, and total dry solids were observed after the experimental challenge, remaining constant before, during, and after therapy. Considering the relevance of routine antimicrobial treatment and the growing interest in natural alternatives, the results demonstrated comparable effectiveness between the two therapy approaches, particularly in preserving the main components of milk [18]. More and more scientists are busy searching for the bactericidal properties of various plants and other substances [19–24], including to combat against causative agents of goat mastitis due to the multi-resistance of some microorganisms [25–27].

In Norway, SCC in bulk milk has been registered at the level of more than 1 million/ml in recent decades, and in healthy goats, according to the country’s requirements, it should be less than 500 thousand/ml. A report from the Goat Health Registry shows a mastitis incidence of 2%–3%. This figure is underestimated because farmers cull sick goats or leave them untreated [28].

Intramammary infection can be caused by different pathogens. While low-pathogenic pathogens are more often associated with a mild clinical course of mastitis, highly pathogenic pathogens cause more severe inflammation and an increase in the SCC [8].

A universal threshold indicator of the SCC, changes in the physical and chemical composition for differentiating milk from a goat with subclinical mastitis and a healthy goat, does not yet exist [13]. Indirect studies suitable for establishing cutoff values that indicate subclinical mastitis have not yet been found. The aim of this study was to evaluate the index of chloride content in goat milk as a probable parameter related to subclinical mastitis and to determine the influence of subclinical mastitis on milk composition.

## Material and Methods

### Ethical Approval

Studies on goats were agreed upon with the Commission for Bioethical Expertise of the University, in accordance with the international standards set by the European Convention “For the Protection of Vertebrate Animals Used for Experimental and Other Scientific Purposes” (WorldLII, 1986).

### Goat Milk Sampling

Individual milk samples from 27 German white and Alpine goats of one farm in the Dnipropetrovsk region, Ukraine, were studied. The system of keeping goats is stall-walking. Feed and water were available to the animals at all times. All goats were clinically healthy. Goats of the second–fourth lactation, milk yield 1–3 L. Goats were milked twice a day with a milking machine. An average milk sample was taken from each goat after morning milking. For bacteriological examination, samples of milk were taken in sterile test tubes at the beginning of milking, treating both teats with a cotton swab with alcohol. The milk was transported within 2 h to the laboratory in a refrigerator bag at a temperature of 4°С ± 2°С.

### Biochemical Analysis of Milk

The composition of milk was studied at the Parasitology and Vet Expertise Department of the University. The main milk indicators were studied on an ultrasonic analyzer Ekomilk Milkana Kam 98–2a (Bulteh, Bulgaria). Limits of permissible absolute error when measuring the mass fraction of fat (± 0.1%), protein (± 0.15%), and freezing temperature (± 0.01°С).

A settling test with milk and a rapid mastitis test (an analogue of the CMT) were carried out—a test with mastidine (Reagent, Ukraine). A titrimetric method was used to determine the chloride ion concentration in milk. The calculation of the chlorosugar number was made.

The SСС was determined using a viscometric analyzer, “Somatos” (Kostil, Russia). The principle of operation of the device is based on the change in viscosity—the time of outflow through the capillary of the milk sample, which is mixed with the drug “Mastoprim” (Reagent, Ukraine), depending on the SCC. The relative error of conventional viscosity measurement is no more than 5%.

After determining the main indicators, milk samples were separated into three groups depending on the chloride ion concentration: group I < 70 mmol/l; group II 70–85 mmol/l; and group III > 85 mmol/l.

### Preparation of Milk Smears

**Figure 1.**
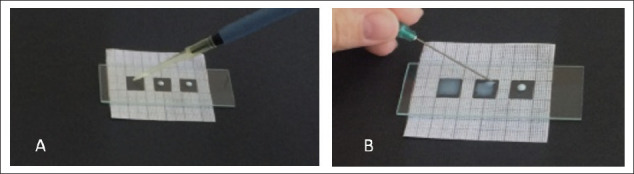
(A) Application of 0.005 ml of milk on slides using a micropipette, (B) the process of making smears of goat’s milk.

Smears were also made from all goat milk samples. Milk smears were made using our own improved technique using a stencil with cut-out squares of 1 cm² on millimeter paper. A sample of milk (0.005 ml) was applied with a micropipette to the slide (Fig. 1A). Then the milk was evenly distributed with a needle over the entire area of the square (Fig. 1B) Then the smears were dried, fixed, and stained by two methods: with pyronin Y and methyl green, according to May-Grünwald. Somatic cells were counted according to the Prescott and Breed method under a microscope, with 100 objective magnifications [29].

### Bacteriological Studies of Milk

A bacteriological examination of milk was carried out at the Biosafety Center of the University. The total plate count was determined by inoculating 1 ml of the prepared test material on meat peptone agar followed by incubation at a temperature of 30°С ± 2°С for 72 h. Post-incubation, the colonies were counted, and the CFU/ml of the sample was assessed. To determine the pathogens, a primary culture was carried out on an enriched liquid medium—heart–brain broth. Cultures were carried out on meat peptone agar with 5% of the erythrocyte mass of sheep blood, salt agar, Endo agar, and Saburo agar with dextrose and chloramphenicol. The biochemical properties of the selected microorganisms were determined using tests (Analytical profile index—a system for rapid identification of known microorganisms) (BioMerieux, France). Catalase activity was determined in the reaction with hydrogen peroxide. The hemolytic properties of cultures of microorganisms were evaluated by the zone of hemolysis on blood agar (α-, β-, or γ-hemolysis). To identify streptococci, the CAMP test (named after the inventors Christie–Atkins–Munch-Petersen) was also performed on blood agar with a reference strain of *S. aureus*.

### Statistical Analysis

Data were analyzed using Microsoft Excel and Statistica 12 (StatSoft) computer software. Results are expressed as mean ± standard error (x ± SE). Statistical differences between groups were evaluated using Tukey’s post hoc test, with significance accepted at *p* < 0.05, adjusted using the Bonferroni correction.

## Results

The number of chlorides in cow’s milk ranges from 25 to 66 mmol/l [30]. Chloride levels in milk rise sharply in response to animal diseases, notably mastitis. There is no data on the amount of chloride in the milk of goats with subclinical mastitis.

After determining the main indicators, milk samples were separated into three groups depending on the chloride ion concentration: I group < 70 mmol/l; II group 70–85 mmol/l; III group > 85 mmol/l. The content of chloride ions differed significantly between groups of milk samples (*р* < 0.05). If the content of chloride ions is > 85 mmol/l, it is suspected that the milk was collected from a goat affected by subclinical mastitis, because in this group the highest number of somatic cells, positive tests with mastidine, and the settling test were noted. The causative agents of mastitis were also detected.

Some other indicators of milk also changed significantly depending on the group to which they belong (Table 1).

**Table 1.** Table 1. Composition of goat milk depending on chloride level (x ± SE).

Parameter	Groups of milk samples by chloride content, mmol/l		
	І (n = 8)	ІІ (n = 13)	ІІІ (n = 6)
The content of chloride ions, mmol/l	63.01 ± 1.32^a^	76.17 ± 1.07^b^	97.01 ± 4.17^c^
SCC, × 103 cells/ml: "Somatos"	439.0 ± 159.3^a^	1,672.6 ± 292.2^b^	2,500.5 ± 316.0^b^
May-Grünwald method	634.2 ± 169.0^a^	1,569.4 ± 323.4^b^	2,149.1 ± 560.3^b^
pyronin Y method	703.2 ± 213.4^a^	1,484.0 ± 276.2^b^	2,273.4 ± 539.3^b^
Fat, %	4.35 ± 0.74	4.28 ± 0.47	4.75 ± 0.62
Solid-not-fat, %	8.20 ± 0.27	8.13 ± 0.10	8.86 ± 0.34
Protein, %	3.06 ± 0.09	3.02 ± 0.04	3.30 ± 0.12
Lactose, %	4.52 ± 0.16	4.48 ± 0.05	4.80 ± 0.24
Freezing temperature, °С	–0.540 ± 0.015	–0.533 ± 0.006	–0.579 ± 0.023
Density, °А	27.21 ± 1.40	27.02 ± 0.43	29.60 ± 1.71
Electrical conductivity, mS/cm	4.73 ± 0.26^a^	5.35 ± 0.13^b^	6.21 ± 0.46^b^
pH	6.71 ± 0.04	6.67 ± 0.04	6.90 ± 0.16
Chlorosugar number	5.00 ± 0.23^a^	6.05 ± 0.14^b^	7.21 ± 0.27^c^
Bacterial contamination, CFU/ml	1.1 ± 0.24 ×105	9.0 ± 4.9 × 106	1.6 ± 0.5 × 106
Pathogens of mastitis	–	–	+
Mastidine test	–	+	+
Settling test	–	–	+

Note: different letters in the same row indicate selections that show significant differences from each other according to the results of the Tukey test (*p* < 0.05) with Bonferroni correction. If there is no letter above the numbers in the row, it means that there is no significant difference between any selected parameters.

According to previous research, it was found that during direct somatic cell counting in goat milk smears stained by various methods, a greater number of cells was determined than with the help of devices [31]. Based on this, to objectively determine the SCC, this indicator was measured by the hardware method and the arbitration method of direct counting of cells in smears of milk (according to the Prescott and Breed method), stained in two ways. SCC indicators determined by three methods did not differ much within the same group (Table 1). SCC is such a variable indicator that an error of 15% is considered the norm for hardware methods.

SCC, which was determined by direct counting in smears and with the help of the viscometric analyzer “Somatos,” in group III is 3.2–5.7 times greater than in group I, depending on the research method (*р* < 0.05). The difference between the indicators of the II and I groups is also significant (*р* < 0.05). SCC in milk samples of group III is 1.4–1.5 times (depending on the research method) higher than that of group II, but no statistical difference was found.

The parameter of conductivity of samples of the II group is 1.13 times higher and III is 1.31 times higher in relation to milk of the I group (*р* < 0.05).

There is a certain ratio between the amount of lactose and chlorine, which is called the chlorosugar number. In the milk of healthy cows, this indicator does not exceed 4, and in the milk of cows with mastitis, it reaches 10–15 [30]. There are no data on the chlorosugar number of goats’ milk.

According to the results (Table 1), this indicator in healthy goats with chloride content < 70 mmol/l in milk was 5.0 ± 0.2 (from 4.1 to 5.9 in the group of animals). In the group of goats with chloride content > 85 mmol/l in milk, the chlorosugar number was 7.2 ± 0.3 (from 6.5 to 7.9) (*р* < 0.05). The chlorosugar number in the milk of goats of III and II groups is 1.4 and 1.2 times higher than that in group I, respectively (*р* < 0.05). Moreover, goats in group III showed a 1.2-fold increase in this parameter compared to group II (*p* < 0.05).

An increase in the chloride content of goat milk is associated with a rise in bacterial contamination, but in the II group of milk samples, it is 5.7 times more than in the III. No statistical difference between the indicators was found due to large average statistical deviations.

Thus, according to the results of research, the bacterial contamination of milk does not always correlate with the number of somatic cells. A quick mastitis test was conducted—the mastidine test. With the milk of goats of the I group, the result was negative; II and III were positive.

During the settling test, a sediment or a clot was detected at the bottom of the test tube only in the milk of goats of the III group. This gives reason to claim that a positive result of the reaction is noted for chloride content > 85 mmol/l. We found out some features of accounting for the settling test with goat’s milk. It is better to record results after 24–36 h. It is necessary to take a good look at the test tube with milk—in case of subclinical mastitis, the milk is watery (with a grayish tint) in the lower half of the column, or there is barely noticeable sediment or clot at the bottom of the test tube. If you pour the contents of the test tube into a Petri dish, everything mixes into a homogeneous mixture, but a trace of a clot remains on the glass of the test tube. In the absence of subclinical mastitis, the test tube contains normal milk with a layer of cream.

Bacteriological analysis of goat milk for mastitis pathogens revealed *Streptococcus agalactiae* in 2 out of 6 examined samples (group III).

With chloride content > 85 mmol/l, an increase in protein, fat, solid-not-fat, lactose, and pH was found compared to the first group (Table 1). The freezing temperature, on the contrary, decreased by 7.2%, but no statistical difference was found.

## Discussion

Somatic cell count, the most informative indicator for diagnosing udder health in cows [32,33], may not be a reliable parameter for determining subclinical mastitis in goats. This indicator changes a lot (for example, during the lactation period of goats), and often the value of more than 1 million/ml is determined without the appearance of subclinical mastitis. An increase in somatic cell count can result only from physiological factors (e.g., breed and stage of lactation), hygiene conditions, and the use of milking equipment [7,9].

Determining the bacteriological status is the gold standard for detecting mastitis, but it requires significant time for analysis, it is expensive, and it is not often used. It is proposed to use an alternative parameter to diagnose subclinical mastitis in goats, such as the SCC, electrical conductivity, changes in milk components, N-acetyl-β-Dglucosaminidase and others [13].

Although conductivity measurement is widely used to determine udder health in cows, it is not commonly used in small ruminants [34]. Schüppel and Schwope [35] determined the average electrical conductivity to be 6.6 ± 0.5 mS/cm. These values do not align with the results of our study, in which the electrical conductivity of milk from healthy goats was 4.73 ± 0.26 mS/cm. However, a definitive threshold for distinguishing between infected and non-infected mammary glands in goats has not yet been established [34]. Therefore, a significant correlation between electrical conductivity and somatic cell count in dairy goats appears to be lacking.

No direct association between the SCC and bacterial contamination of goat milk was found. Subclinical mastitis does not show any external symptoms of inflammation, except for an increase in the somatic cell count and the presence of pathogenic microorganisms in milk [13]. Kyozaire et al. [36] also note the absence of a probable dependence of SCC on the presence of microorganisms in goat milk.

It is well established that SCC, even in a healthy udder of goats, is much higher than that of cows due to the apocrine type of secretion. A positive CMT mastitis test requires confirmation by bacterial testing [37].

Conducting bacteriological research on milk samples is considered the “gold standard” for assessing udder health, but bacteriological tests are too expensive and time-consuming. Therefore, indirect methods of detecting udder health and bacterial infection are being sought [13,38,39].

Threshold value—an indicator of subclinical mastitis in a cow’s bulk milk 400 × 10^3^ cells/ml in milk is impractical for determining subclinical mastitis in goats. Factors such as breed, multifertility, and period of lactation provoke essential changes in the SCC. The number of somatic cells is also affected by a low- or high-pathogenic pathogen in goat milk. Some authors even claim that the SCC is not suitable as a parameter for monitoring mastitis in goats. For example, Silanikove et al. [40] came to the conclusion that coagulase-negative staphylococci and the lactation period are the two most important reasons that worsen the safety of milk. Bacterial infection and milk from late lactation are associated with a positive correlation between casein, lactose, and curd firmness, and a negative correlation between curd firmness and SCC.

The majority of scientists definitely attribute the signs of subclinical mastitis in goats to a large SCC in milk in combination with mastitis pathogens [1,6,10]. In the present study, out of six samples collected from group III, the mastitis pathogen *S*. *agalactiae* was isolated in two. In my own research, it was by these criteria that subclinical mastitis was determined in goats of the III group, and a positive settling test was also taken into account. Goats of the III group have the highest content of chloride ions, which is why this indicator can be considered a probable sign of subclinical mastitis.

According to the results of their own research, with a chloride content > 85 mmol/l, an increase in protein by 0.24%, fat by 0.4%, solid-not-fat by 0.66%, lactose by 0.28%, and pH by 2.8% was found, compared to the first group (Table 1). The freezing temperature, on the contrary, decreased by 7.2%, but no statistical difference was found. The results of my own research coincide with the data of other scientists. Thus, Leitner et al. [41] found no significant difference in fat content between infected (3.75%) and non-infected (4.2%) udder lobes in samples from 10 different herds. These results were validated by studies of 35 Alpine goats of different ages [42]. There is no significant relationship between infectious status and milk fat content.

Only experiments by a team of researchers, Ying et al. [43], showed a significant relationship of pathogens in milk samples, expressed as the logarithm of total microbial contamination, associated with fat concentration during early lactation.

Regarding the influence of subclinical mastitis on the protein content, Leitner et al. [44] determined a higher content of protein in milk from an infected than from a non-infected udder (3.50 ± 0.05 *vs.* 3.42% ± 0.05%), but without statistically significant differences. Significantly higher concentrations of lactose were found in healthy than in infected udders of goats (4.96% *vs.* 4.72%) [42]. Goats with subclinical mastitis had lower levels of fat, lactose, and milk yield, but protein content was slightly higher compared to healthy goats [45]. An increase in the content of protein, fat, and solid-not-fat in the milk of goats with subclinical mastitis is reported by Peixoto et al. [18], which is consistent with our results.

## Conclusion

High chloride ion levels in milk may indicate subclinical mastitis in goats because it is combined with a large SCC, the presence of mastitis pathogens, a positive settling test, and a mastidine test. It was also found that with subclinical mastitis, the electrical conductivity and the chlorosugar number probably increase. A set of indicators, such as chloride content > 85 mmol/l, the number of somatic cells > 2 million/ml, and chlorosugar number ≥ 7, along with confirmed results in the settling and mastidine tests, can be used as a diagnostic criterion for subclinical mastitis in goats. Further research will be aimed at preventing subclinical mastitis in goats by using udder sanitation with new (plant-based) agents.

## List of abbreviations

API tests, Analytical profile index

CAMP test, Christie-Atkins-Munch-Petersen test

CFU/ml, colony-forming units per milliliter

CMT, California mastitis test

PCR, polymerase chain reaction

SCC, somatic cell count

ml, milliliter

L, liter

h, hours

°C, degree Celsius

mmol/l, millimoles per liter

mS/cm, milliSiemens/centimeter.
